# Reflections on augmented reality codes for teaching fundamental defensive techniques to boxing beginners

**DOI:** 10.1371/journal.pone.0301728

**Published:** 2024-04-11

**Authors:** Ahmed Hassan Rakha

**Affiliations:** 1 Faculty of Physical Education for (Men–Girls), Department of Curriculum and Teaching Methods of Physical Education, Port-Said University, Port-Said, Egypt; 2 Department of Physical Education and Kinesiology, College of Education, Qassim University, Buraidah, Saudi Arabia; Tsinghua University, CHINA

## Abstract

AR technology allows users to interact with virtual objects in real-world settings. Immersive AR experiences can enhance creativity and possibilities. Learners can explore real-life situations in a safe, controlled environment, understand abstract concepts and solve problems. This study investigates whether AR-codes affect boxing beginners’ performance in some fundamental defensive techniques. An experimental and control group were created to implement a quasi-experimental design. By using the ASSURE instructional design model, AR technology was incorporated into the educational program and delivered in flipped classroom method to the experimental group. Comparatively, the control group is taught a program using a teacher’s command style. A post-measurement of defensive boxing skills was conducted for both groups. Participants were 60 boxing beginners aged 12 to 14 who had enrolled in Port Fouad Sports Club’s 2023/2024 training season in Port Said, Egypt. Randomly, participants were divided into control and experimental groups. They were homogenized and equivalent in terms of age, height, weight, IQ, physical fitness, and skill level. According to the study results, the experimental group performed better in post-measurements than the control group. The AR Codes technology had a large effect size on the learning of boxing defensive skills under study. Consequently, it is necessary to use AR Codes technology as an educational resource to enhance the learning process, integrate it with active learning strategies, and use it to teach defensive boxing skills and apply them to offensive and counterattack skills, thereby improving the learning process.

## Introduction

In augmented reality (AR), users interact with virtual objects in the real world by combining digital and physical information through various technologies, such as smartphones. Virtual objects appear in the same space as real objects, allowing for the display of 2D and 3D virtual objects in real-time [1–4]. AR is not only used for professional purposes, but also for entertainment and leisure [5]. Becker et al. [6] indicated that AR offers new learning and teaching opportunities and is expected to have a future in K-12 education, so teachers should be aware of its potential. AR software relies heavily on mobile technologies, making it a form of mobile learning [7].

Through AR, we can experience situations that would be near impossible in the real world by combining physical and virtual objects, as well as make abstract concepts more concrete and accessible [8,9]. AR technology can be used to create immersive learning environments that facilitate interactive and collaborative learning, as well as authentic and situated learning [10]. Previous studies have shown that AR improves student attitudes and satisfaction levels, increases motivation to learn, and leads to better academic outcomes [7,11–14].

Additionally, its high level of surprise makes it a highly motivating resource [15]. Studies have also shown that learners who use AR have higher levels of learning enjoyment, interest, and collaboration, as well as more enjoyable learning experiences and student-centered teaching environments [3,16–18].

A Quick Response (QR) code is a two-dimensional barcode decoded by QR scanners and smartphones. QR codes can contain contact information, SMS messages, plain text, or URLs [19]. As QR codes are used in a wide range of educational activities, they enable direct connections between printed materials and online resources. As part of the learning mobile, teachers can choose or develop digital resources based on the students’ age and ability level, which can be filtered according to their learning capabilities [20]. In fact, this feature makes them integral to AR learning [21].

Boxing, like other combat sports, relies on physical attributes, technical proficiency, tactical awareness, and mental toughness. Mental preparation is the most significant factor for novices to improve their physical, technical, and tactical performance. Cognitive awareness is essential for the development of psychomotor abilities, spatial awareness, and learning motivation. For enhancing education and training programs, it is essential to comprehend boxing techniques in order to attain optimal motor skills [22,23]. Boxers who master the fundamental skills of boxing are much better at linking attack, defence, and counterattack.

Integrating and using technology in education requires knowledge of the learning process and how it can be incorporated. Additionally, it involves selecting the right instructional design (ID) model for providing standardized education that meets the needs of learners, introducing technology, and designing educational activities. To incorporate AR technology into teaching defensive skills in boxing to beginners, a theoretical framework will provide insights into the use of AR in education. Mobile learning will be the technology used for this integration, allowing boxers to interact with the AR technology. The flipped classroom strategy will help deliver and organize the utilization of AR technology in learning. Additionally, the ASSURE model will be utilized to ensure an effective integrated educational program. This model involves analysing learners, defining educational objectives, developing the technological medium, and determining teaching and evaluation strategies.

## Theoretical framework

### AR in education

#### AR definition

AR has done a good job of facilitating student learning and encouraging them to engage in meaningful and enriching experiences. In this educational technology, the user is able to gain access to and expand their knowledge of the environment where they are located with the help of a mobile device [24].

The Oxford Dictionary defines the term ’augment’ as ’to make something better by adding to it’. The concept of making greater refers to enlarging, extending, or increasing the features of physical components. The use of AR enhances physical reality by using digital components [25].

#### AR types

**Fig 1:**
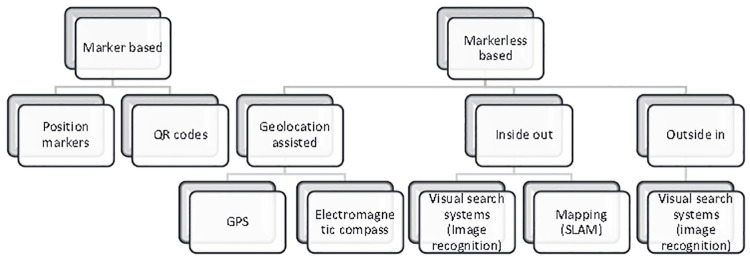
AR types. AR consists of two major types: Marker-based AR systems and markerless AR systems. Marker-based AR systems utilize fiducial markers or graphics to activate digital output and display virtual content. On the other hand, markerless AR systems are more common and typically employ optical-mechanical, ultrasonic, magnetic, or inertial sensors to recognize objects, patterns, shapes, and locations.

According to the literature, AR types can be categorized according to the technology employed, the type of interaction, and the recognition features provided. As shown in Fig 1, these classifications have been systematically combined for greater clarity [25].

1. A marker-based AR system employs printed fiducial markers or graphics to activate digital output and display the related virtual content. After scanning the marker, digital outputs can include 2D or 3D photos, films, animations, or audio. Marker-based systems have three major components: (1) a printed marker or visual information, (2) a gripper for displaying digital content (e.g., camera), and (3) augmented digital content presented on a screen.
  - Position markers for marker-based AR use a camera and visual markers to determine the centre, orientation, and range. The virtual output is orientated at the center of the marker, and the digital output can be viewed from different perspectives by reorienting or rotating the marker [26–29].
  - AR systems that use QR codes function similarly, though the digital content is linked to and recalled directly through the QR code. QR-code AR users can access digital content regardless of the content’s inability to be rendered onto the virtual layer. Therefore, QR-code AR doesn’t require rotation or orientation[25]. Current study will be relying on marker-based AR system for teaching boxing defensive techniques.
2. The markerless AR system is more complex and more widely applicable than marker-based AR since the fiducials are not needed. Most of these systems recognize objects, patterns, shapes, or locations using optical-mechanical, ultrasonic, magnetic, or inertial sensors. Digital content is then displayed on a display system [29].

In the current study, boxing defensive techniques will be taught using a marker-based AR system.

#### The role of AR in education

Wu et al. [30] classified three major categories for approaches that use AR in education:

1. Emphasizing roles: Participants in an AR environment were encouraged to take on different roles through participatory simulations, role-playing, and jigsaw puzzles. As these approaches emphasize collaboration and interaction among students, they are commonly associated with mobile AR, multiplayer AR, or game-based AR [30]. In a participatory simulation, different players function as interacting components of a dynamic system, which affects the outcomes of the simulation [31]. Studies such as [32–34] have recommended that AR simulations can engage students, especially those who had previously presented behavioural and academic challenges to teachers. Role-playing simulates real-life situations, allowing students to practice decision-making, problem solving, and communication skills in a safe setting. Additionally, it encourages them to think critically and develop empathy[35]. In some AR environments, students play different roles to gain a deeper understanding of a topic. As an example, in Squire and Jan [36], students portrayed environmental investigators, scientists, and environmental activists in order to gain a deeper understanding of scientific investigation’s social context. In addition to participating in a simulated system, students also gained access to information relevant to their roles or adopted different ways of thinking while playing each role. Furthermore, students can complete tasks through role-playing using a jigsaw approach that emphasizes collaboration between different roles [30,32].
2. Emphasizing location: Using mobile-AR with location-registered technology is a common subset used in place-based or location-based learning to emphasize learners’ interactions with the physical environment. These AR environments take advantage of mobile technologies because they can track learners’ actual geographical location through their devices [30]. Students may feel more authentic when they engage in place-based learning. Working in a physical area or moving through an actual environment may help students feel more grounded in "reality" [37,38].
3. Emphasizing tasks: A variety of approaches to learning are available, such as game-based, problem-based, and studio-based methods. The varied nature of the tasks does not require the implementation of a specific subset of AR technologies [30].

According to Yuliono and Rintayati [39], AR has three important roles in educational settings: learners’ outcomes, pedagogical contributions, and interaction:

1. *Learner Outcomes*: AR had the greatest impact on the learner outcomes by increasing students’ knowledge and understanding in a variety of subjects [40–45]. It also helped students to improve their skills [46–51]. In addition, it enhanced students’ motivation, learning effectiveness, satisfaction, and achievement in the classroom [3,52–56].
2. *Pedagogical contributions*: AR helps teachers engage their students during the learning process. According to Akçayır and Akçayır [3], AR raised engagement levels. Additionally, Kamarainen et al. [57] found that AR enhanced probeware-based activities on a field trip by engaging, structuring, and organizing them. Students can also benefit from AR because it facilitates centered learning. In addition, AR transformed teaching and learning into student-centered activities [58–60]. Moreover, AR helps students learn independently by allowing them to access resources independently. Furthermore, AR made material delivery more efficient. By using AR as a tool for delivering the materials, students were able to experience authentic simulations and vivid visualizations [47,61].
3. *Interaction*: In the teaching and learning process, AR improved interaction. Akçayır and Akçayır [3] found that AR promoted interaction between students, between students and materials, and between students and teachers. Frost et al. [62] concluded that AR clearly enhanced constructivism in teaching and learning.

### Quick response (QR) codes for delivering AR contents

**Fig 2:**
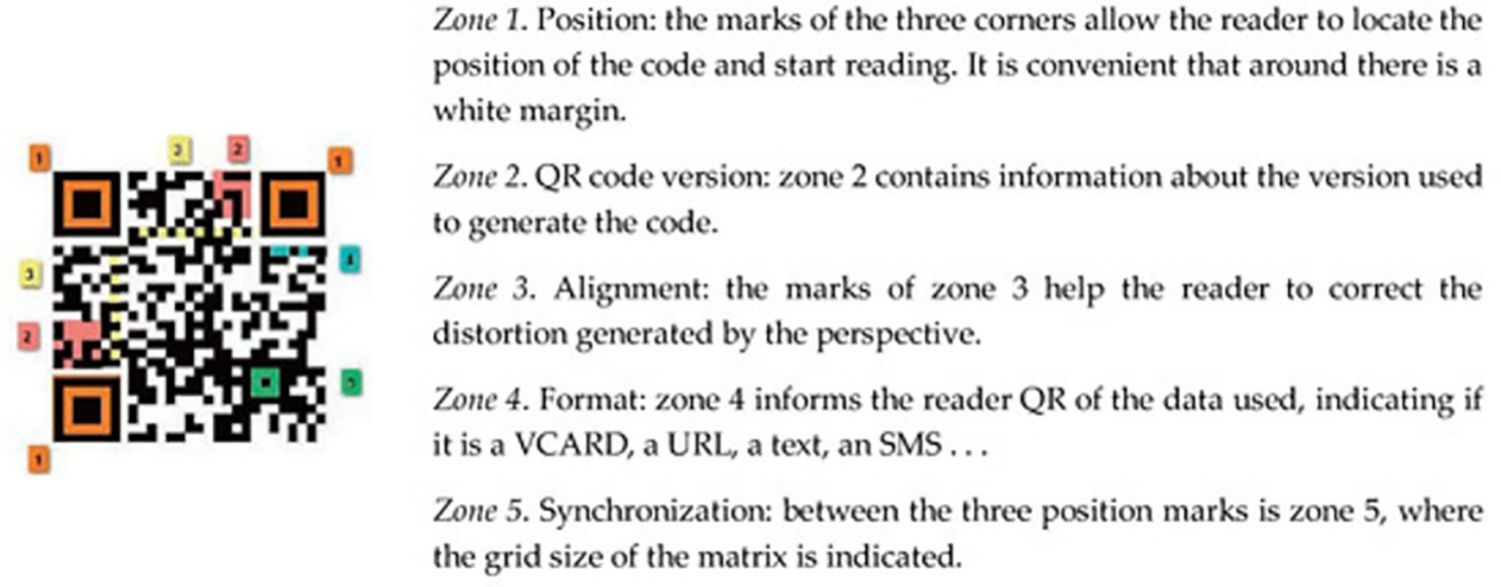
QR code functionality description. In QR codes, the reader can detect the position of the code by using the three squares in the corners.

According to Ortega-Sánchez and Gómez-Trigueros [63], QR-Codes encode vast and diverse information in a matrix of dots. They are identified by the three squares located in the corners. This allows the reader scanner application to discover the stored information, as shown in Fig 2 [63,64]:

In the context of mobile learning, QR codes are employed in the educational setting. By scanning QR codes for data such as text, URLs, or other information, students can have access to resources associated with a certain topic or place. Users may display text, visit a web page, send automated SMS messages, and more by scanning QR Codes with a smartphone connected to the internet and a QR Code reading app. Numerous free apps may be used to decode and read QR Codes, and it can even be incorporated into educational games [65,66].

According to a number of studies [67–69], QR codes are an effective tool for bridging the gap between printed and multi-media educational resources in formal education, as well as improving learning outcomes, learning effectiveness, and learners’ attitudes toward education.

According to Del Rosario-Raymundo [70], QR code technology for education has a number of advantages: adaptability to various learning styles; enhanced educational opportunities; provision of social activities; encouragement of learner involvement; the provision of feedback to students with various educational needs; as well as unlimited access to information and a reduction in frustration and a rise in independence.

QR codes, according to Law and So [19], fulfil three AR learning criteria: location independence, time independence, and meaningful content.Location independence refers to learning occurring in various settings, both indoors and outdoors, formal and informal, without being limited to specific locations. Time independence refers to learning outside of the classroom. Meaningful content is versatile and suitable for learners in a variety of contexts, making QR codes a useful tool for helping learners learn through AR.

Using QR Codes as the traditional AR marker, AR based on QR Codes extracts and displays information from QR Codes in a 3D format. In conventional AR systems, special markers determine the 3D structure of the scene and which objects should be displayed. QR Code markers are used only for tracking and identification, and no other information is conveyed. By scanning the QR code that is pasted on the printed scientific material, a 3D virtual object is displayed in the real world. This system allows learners to visualize and interact with educational content directly through AR technology [71].

### Mobile Learning (ML)

As defined by Geddes [72], ML consists of learning any knowledge or skill through mobile technology anywhere, anytime, resulting in behaviour modification. In addition, ML might be generally defined as any educational system that uses handheld or palmtop devices exclusively or mostly. This definition includes smartphones and their peripherals, tablet PCs, and laptop computers, but it excludes desktops and other similar devices [73].

Several issues are unique to ML environments [74]:

1. *Need for urgent learning*: If learners need to learn anything right now, they can utilise wireless apps (such as those that link problem solving and knowledge), or they can record their queries and look up the answers in the library, online, or from a specialist. Additionally, learners can watch tutorials or educational videos, or join online courses and webinars. Social media such as forums and blogs can also be used for learning, as well as attending workshops and seminars.
2. *Knowledge acquisition initiative*: In a timely manner, a wireless application can provide relevant information based on a learner’s request (e.g. information on demand).
3. *Mobilized learning environments*: As wireless devices become more portable, the educational practice can occur anywhere and at any time (e.g. on a trip, a camping area, etc.). The possibilities of field trips are endless.

ML can use AR technology as one of its possibilities [75]. A study by Kamarainen et al. [57] shows that mobile technology and AR enhance content learning in real-life environments. The combination of AR with mobile devices can influence learning experiences as well as motivation [76,77]. By incorporating AR into ML, students learn skills and professional competencies faster and with less effort [78].

### Flipped classroom

A Flipped Classroom brings the practical parts of the class into the classroom (e.g., activities and problem solving), which are usually done outside the classroom. Instead, what used to be done in class (e.g., information presentations and information transmissions) is now done outside of the classroom [79]. With Flipped Classrooms, interactive learning activities take place during class and individual instruction takes place outside of class. They are active learning approaches when used correctly.[80].

In order to flip a classroom, four essential pillars need to be met. According to the initial letters of the "Flip" word, this strategy has the following pillars:

1. **F**lexible Environment: indicates that learning can take place at any time and at any location.
2. **L**earning Culture: In the traditional teacher-centered approach, the teacher is the source of knowledge. Flipped classrooms transition from a teacher-centered approach to a student-centered approach. Therefore, students participate in and evaluate their learning in a meaningful way, therefore actively constructing knowledge.
3. **I**ntentional Content: Students should handle materials on their own as part of flipped learning. The educator determines what needs to be taught and what students should handle themselves. To maximize classroom time, educators use Intentional Content, depending on grade level and content, in order to adopt student-centered, active learning strategies.
4. **P**rofessional Educator: Flipped classroom educators have more responsibility than traditional educators do. The educator in a flipped classroom constantly observes the students, evaluates their work, and provides feedback to them during the course [81,82].

According to several studies on flipped classrooms, this strategy stimulates active learning during classroom contact hours and, on the other hand, encourages independent learning outside of the classroom. As a result, it improves social interactions and promotes independent learning. As well, it provided students with the opportunity to learn material at their own pace, according to their own schedule, and according to their own path through online learning platforms before face-to-face learning [79,81,83–87].

### ASSURE model

An instructional environment must be designed systematically according to learning strategies, objectives, and audience abilities in order to achieve desired learning outcomes. These activities are all part of instructional design, or ID. In ID, the primary objective is to create an environment that enhances learning outcomes. ID can be applied in both face-to-face and electronic learning environments. In addition, each environment, technology, topic, and learning strategy indicates different preferences and methods of learning [25]. ID is defined by Koper [88] as the process of teaching and learning within a specific learning object (e.g., a lesson, course, etc.). The ID model represents both the teacher’s and the learner’s learning and support activities within a given learning unit as a whole.

As part of the ID process, learners’ characteristics and environments are investigated, outcomes, methodologies, and assessment tools are developed, educational materials are constructed, learners’ performance is evaluated, and the ID process is evaluated overall [89]. There are many popular and well-known models, such as ID ADDIE, DDD-E, ASSURE, Morrison, Ross, and Kemp, as well as Smith and Ragan. An ASSURE model was used for the current study.

ASSURE is a model developed by Heinich et al. [90] to assist teachers in planning and delivering lessons that incorporate technology effectively. The ASSURE model is popular with teachers because it can be used during a few hours of classroom instruction and for each individual student. There is no need for a deep knowledge of design or a high level of review from the teacher in this model [91].

**Fig 3:**
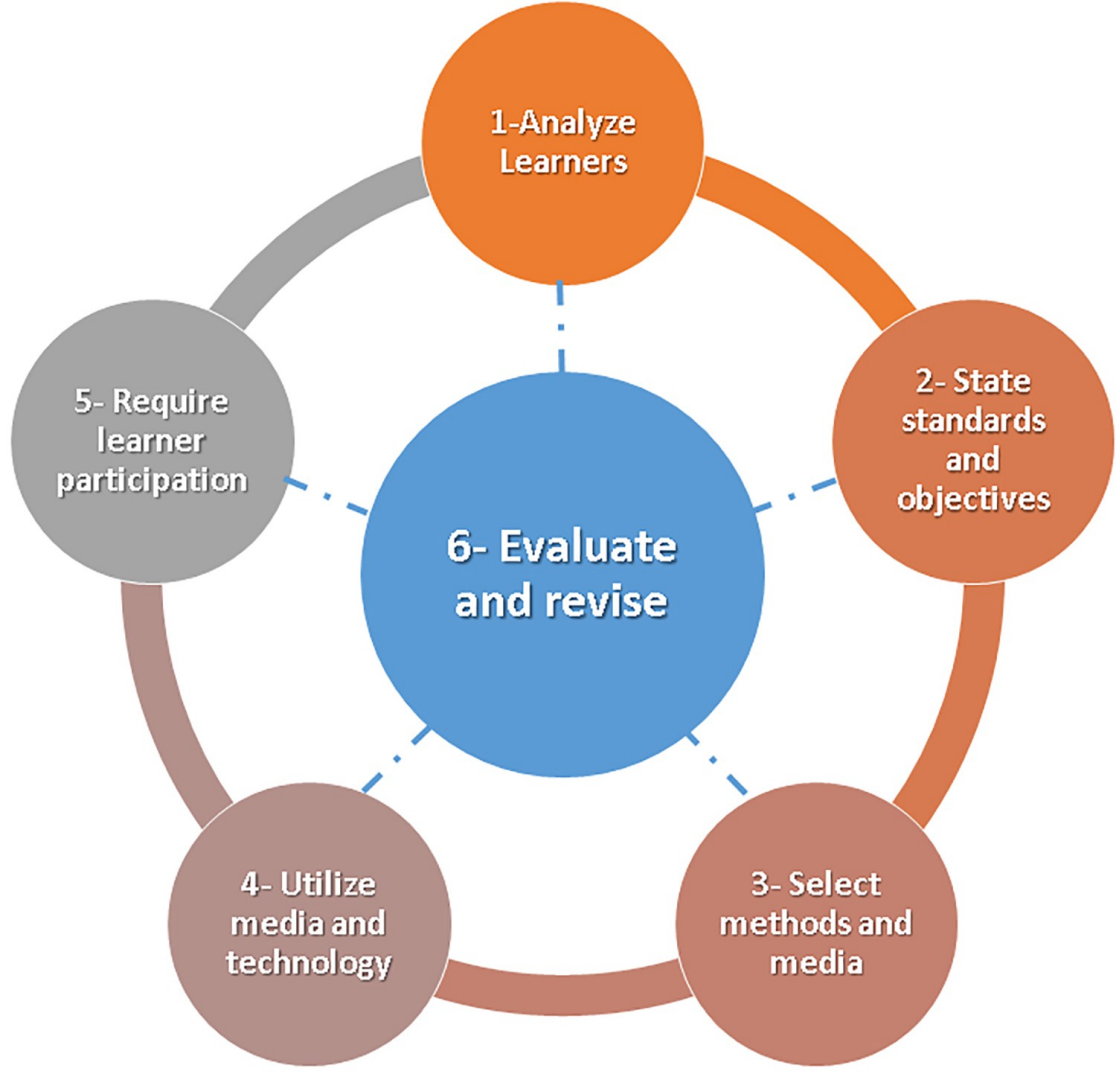
ASSURE model as described by Heinich et al. **[90].** In the ASSURE model, six steps are described by a letter, each of which describes a primary task for making informed decisions about educational technology.

In the ASSURE model, six steps are described by a letter, each of which describes a primary task for making informed decisions about educational technology. According to Fig 3 [90], ASSURE consists of the following components:

1. *Analyze learners*: This stage involves analyzing learners, including their learning needs, objectives, performance gaps, and desired learning outcomes. Moreover, students’ current knowledge, skills, education level, limitations that may affect their learning, and technology options are also considered.
2. *State standards and objectives*: In this phase, the program’s standards and objectives are set.
3. *Select methods and media*: Depending on the content, the third phase involves deciding which media and technology to use. Media and methods should be selected according to who the learners are and where they will learn, as well as how existing materials can be adapted.
4. *Utilize media and technology*: During this phase, educational media and technology are implemented. It also involves mapping, delivering, and providing access to learning technologies.
5. *Require learner participation*: Designing a course should include plans for learners to actively participate in the process. Interactive elements, like discussion forums and collaborative workshops, will enhance learners’ participation and interaction.
6. *Evaluate and revise*: At the end of the ASSURE process, the learning strategies, technology, media, and materials used are assessed for their impact on the instructional program. This evaluation allows us to determine whether the learning objectives have been met, if the technology and materials selected have been effective, and where improvement is needed [90].

Baran [92] showed that students’ progress is facilitated step-by-step using the ASSURE model. Additionally, Rim and Choi [93] found that teachers who design classrooms based on the ASSURE model perform better and integrate technology more effectively. Furthermore, Kristianti et al. [94] found that the ASSURE model significantly improved the critical thinking skills of high school students.

### Research problem

The Amateur International Boxing Association (AIBA) made several significant changes to amateur boxing rules in 2013. These changes prohibited head guards and required 10 oz gloves for boxers weighing 152 lbs, and 12 oz gloves for those over 152 lbs. Each round, the judges must award 10 points to the winner, usually resulting in a 10–9 score for the boxer they believe won. Fouls and other incidents can also be deducted. Before 2013, each bout was judged on the number of landed punches; now, four criteria are considered: the number of quality punches, technical and tactical dominance, competitiveness, and rule violations [95,96].

Due to these changes, coaches have adapted their instruction to encourage boxers to use long-distance punching techniques rather than close-up punching techniques. Additionally, boxers are using footwork defensively, rather than absorbing punches as a defensive strategy, with movement around the ring increasing by 20%. These revised rules have created an environment in which boxers are more concerned about avoiding punches [95,97].

Several boxing studies, including those by [23,98–101] have shown that novice boxers make mistakes that can be addressed and fixed using different instructional scaffolds. According to these studies, instructional films in 3D are the most effective way of raising novices’ understanding of how the skill should be performed.

Due to the revised technical rules and the results of previous studies, this study is essential to bridge the gap between the benefits of AR technology and the requirement for boxers to learn fundamental defensive techniques.

The purpose of the present study is to design and evaluate an educational program based on AR-Codes. Essentially, it is a way to deliver the educational materials to players in a way that engages them, raises suspense, and reveals how it influences their performance in boxing defensive techniques. This goal will be achieved by answering the following question: What are the effects of AR-Codes on boxers’ performance of some fundamental defensive techniques?

According to this main question, the following sub questions emerge: Is there a difference in post-measurement of certain fundamental defensive techniques between experimental and control groups, and what is the effect size of the proposed educational program that incorporates AR codes?

## Materials and methods

### Ethics statement

The Research Ethics Committee at the Faculty of Physical Education, Port-Said University has approved the current study (Approval Number 2023-7-1). A written informed consent was provided for the participants that addressed topics such as their voluntary participation, withdrawal rights, aims, importance, and processes. In the final section of the form, participants can opt to confirm their agreement to participate in the study by selecting Agree or Disagree.

### Design

In this study, experimental and control groups were created in a quasi-experimental design. In the experimental group, AR codes were used to teach fundamental boxing defensive techniques. The control group, on the other hand, is taught a program that is based on the coach’s command style. A pre-post-test design was used for both groups.

### The research hypothesis

*H*_*a*_: There is a statistically significant difference between the control and experimental groups in the post-measurements of some fundamental boxing defensive techniques in favour of the experimental group.

**Fig 4:**
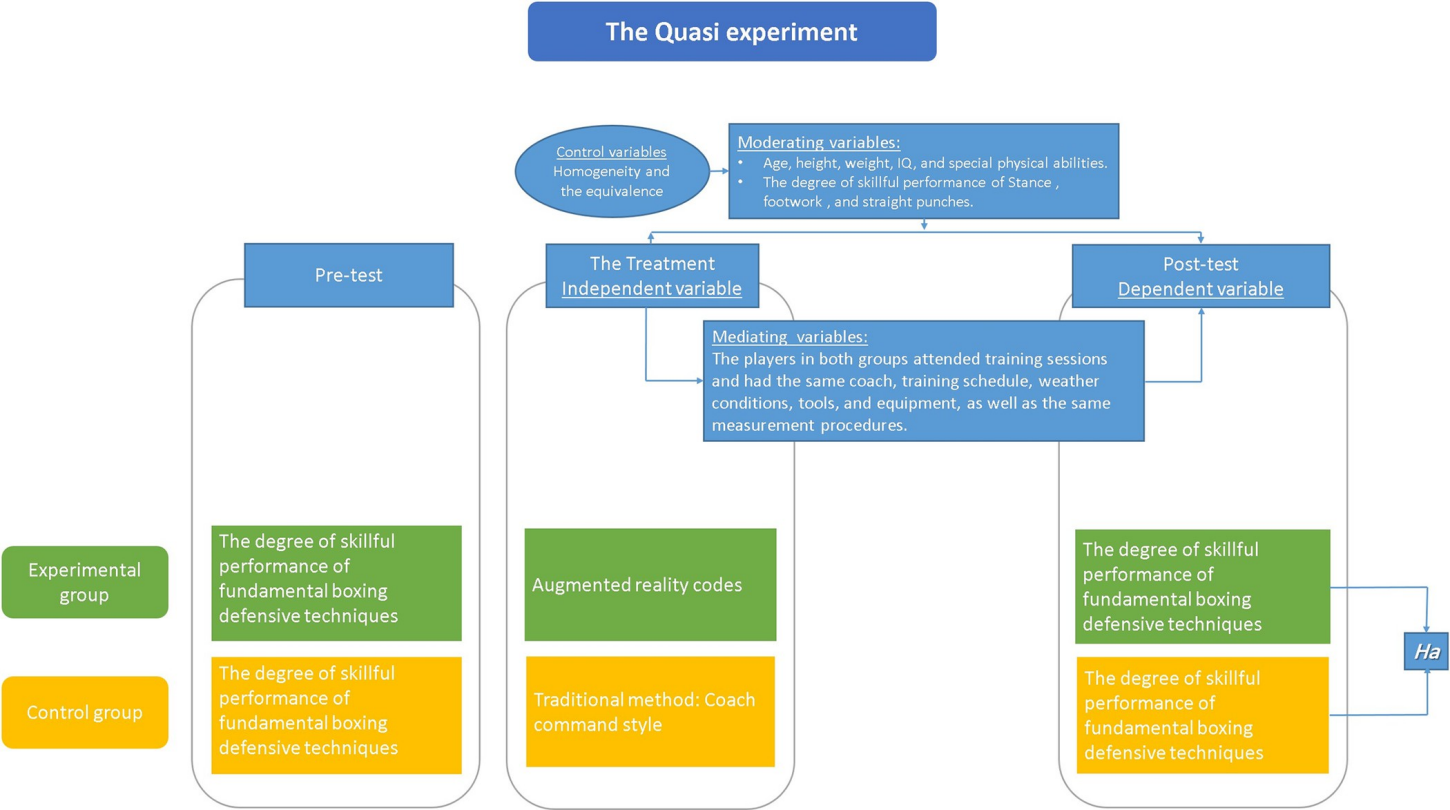
The conceptual framework. Experimental and control groups were created using a quasi-experimental design. The experimental group utilized AR codes to teach fundamental boxing defensive techniques, while the control group was taught using a program based on the coach’s command style. A pre-post-test design was employed for both groups.

A conceptual framework can be seen in Fig 4.

### The priori power analysis

The G*Power 3.1 tool was used for a Priori power analysis [102,103]. By performing a priori power analysis, the sample size for a study is determined according to predetermined Type I, II error rates, and the minimum effect sizes that have clinical, practical, and theoretical validity [104].

The sample size (*n*) is determined by a priori power analysis. This analysis calculates according to the required significance level (*α*), the effect size parameter (*d*) that determined from pilot study or preview similar studies, or we can specify our smallest effect size of interest, the statistical test type, and the desired power (*1-β*) that favours a value of 0.80 or greater [105]. Thus, a statistical test published in the study can be evaluated to see if it had a reasonable chance of rejecting an incorrect H0. Additionally, a priori analyses require *H*1 effect sizes for the underlying population. A Power analysis was conducted to investigate the highly questionable assumption that sample(*n*) effect size is the same as population(*N*) effect size [102].

**Fig 5:**
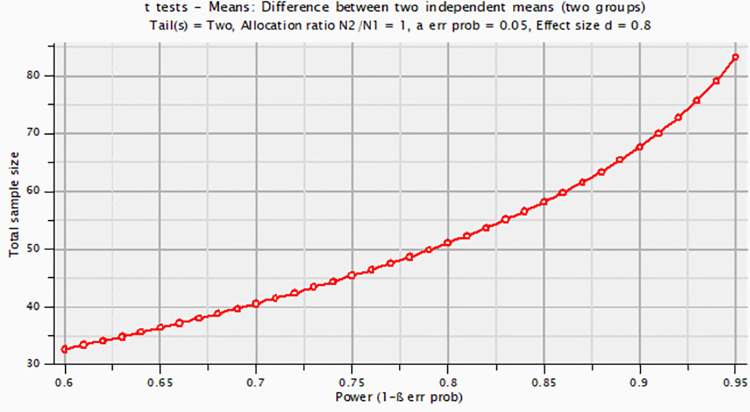
The estimated sample size according to desired effect size. A priori power analysis was conducted using G*Power version 3.1.9.7 software. As a result, the minimum sample size needed for this effect size is N = 52 (n1 = n2 = 26).

As part of the sample size estimation process, an a priori power analysis was conducted using the G*Power version 3.1.9.7 software [102]. Using data from Rakha [23] study (*n*_1_ = *n*_2_ = 20), which compared experimental and control groups. An experimental group is taught boxing skills using a reciprocal style combined with 3D hologram technology, while a control group is taught a teacher-command style. Rakha’s study had an effect size of 1.07, which is considered extremely large based on Cohen [105] criteria. In the current study, on two tails a significance level (α = .05), power *(1-β* = .80), and effect size (*d* = 0.80) parameters were used in G*Power. As a result, the minimum sample size needed for this effect size is *N* = 52 (*n*_1_ = *n*_2_ = 26) for an independent samples *t*-test as shown in Fig 5. Therefore, *N* = 60 is more than sufficient for testing the study hypothesis.

### Study population and sample

Throughout the 2023/2024 training season, 60 beginning boxers aged 12 to 14 participated in the study at the Port Fouad Sports Club in Port Said, Egypt. After the participants were taught straight punches using traditional methods, coach command style, they were randomly divided into two groups by using the random drawing method. This was done to ensure that all members of the group had the same chance of being selected. It was a random process that gave each group an equal chance of being chosen.

**The inclusion criteria:** The participants were homogenized based on their age, height, weight, IQ, fitness level, and skill level in stance, footwork, and straight punches and should be novices in boxing defences techniques.

**The exclusion criteria:** Due to the similarity between some defences’ techniques, such as mixed martial arts (MMA), kickboxing, karate, and taekwondo, the participant must not have any previous experience in combat sports.

### Data collection tools and equipment

1. **A digital stadiometer was used to measure weight and height.**
2. **Test of intelligence quotient (IQ)**
  This study used the IQ test developed by Saleh [106] for measuring mental ability in the sample age group. This test ranks children between the ages of 8 and 17 according to their percentiles and IQ levels. There are 60 homogenous items on the test each containing five images. There are four similar images and one that is different. The participants are given ten minutes to discover the different images. In studies used the test, reliability coefficients range from 0.75 to 0.85, and validity has been confirmed [23,107,108].
3. **Boxing fitness tests for juniors**
  Nine physical fitness tests were administered to participants in the current study. Nasr [109] and Rakha [23] used these tests in their studies involving boxing juniors aged 12 to 14. In these studies, the reliability coefficient for these tests ranged from 0.76 to 0.84, and their validity to distinguish between boxers was confirmed. Testing included the following:
  - *Reaction Time*: The ruler drop test (*cm*).
  - *Kinetic velocity*: Take 10 seconds to punch a heavy sandbag straight left and right (the largest number of punches possible).
  - *The Power of Right straight punch*: Throw a 2*kg* medicine ball as far as possible (*m*).
  - *The Power of Left straight punch*: Throw a 2*kg* medicine ball as far as possible (*m*).
  - *Agility*: by Jumping quadruple in (10 *sec*) test. Two by two m square divided into four equal sections, numbered (1, 2, 3, 4) in a clockwise direction to determine the starting line. Upon hearing the signal, the boxer jumps with both feet together to zone (1), then to zones (2, 3, 4) in order, then returns to zone (1) to repeat the performance until the time is up. (Count of jumps).
  - *Performance endurance*: A two-minute shadow punching record (number of punches).
  - *Muscular Endurance*: Sit up from lying down (The highest number possible).
  - *Muscular Endurance*: Push-Ups (The highest number possible).
  - *Cardio-respiratory Endurance*: Running 1500 meters.
4. **A checklist for arbitrators to evaluate the fundamental skills in boxing**
  A checklist developed by Khalifa [110] contains the following items for arbitrators to evaluate fundamental boxing skills:
  - The guidelines of evaluation criteria for fundamental boxing skills based on technical performance.
  - Guidelines for the phases of fundamental boxing skill evaluation.
  - Skills scoring sheets.

Several previous studies using similar samples have used this checklist.[23,110–112] reported that it had a validity coefficient of 0.97 and a reliability coefficient of 0.87.

### ASSURE model stages for integrating AR-Codes into the educational program

An educational program with AR-Codes was developed in stages based on the ASSURE model:

### 1. Analyze Learners

The study’s participants are between the ages of 12 and 15, in the adolescent period. During this stage, children transition to adulthood. During this period, children’s bodies and brains go through rapid changes as they are exposed to different experiences and develop a sense of identity. Furthermore, brain changes during adolescence increase self-awareness and creativity, while they also begin to form relationships with peers and adults, and learn to make decisions for themselves. As an adolescent reaches physical and psychological maturity, secondary sexual characteristics begin to emerge. A year after the skeletal system develops, the muscular system begins to develop. In addition, rapid growth during adolescence can cause adolescents to become exhausted. Moreover, the torso and legs lengthen, which increases strength and length, along with widening of the shoulders and enlargement of the buttocks [113,114].

### 2. State Standards and Objectives

This program aims to improve some of the fundamental defensive skills of junior boxers between the ages of 12 and 14. According to several studies [110–112,115], boxing juniors should have some fundamental defensive skills listed in Table 1 to defend themselves against straight punches.

**Table 1:** The fundamental defensive skills included in the proposed program.

No	Straight punches	Defensive skills		
		by arms	by trunk	by footwork
1	Left punch to the head	• Block with right hand. • Push to internal with right hand	Leaning trunk to: • Backward. • Right.	Footwork to: • Backward. • Right.
2	Left punch to the Body	• Block by right forearm		Footwork to: • Backward. • Right.
3	Right punch to the head	• Block with right hand. • Push to internal with left hand	Leaning trunk to: • Backward. • Left.	Footwork to: • Backward. • Left.
4	Right punch to the Body	• Block by left forearm		Footwork to: • Backward. • Left.

According to Bloom [116] taxonomy, 102 learning outcomes were developed to fulfil the general objectives of the proposed program: 34 cognitive, 34 psychomotor, and 34 emotional. As an example of the LOs, the following are the LOs of defensive skills against a lead straight punch to the head by using arms:

Cognitive LOs included:

- The boxer mentions defensive techniques by using their arms against a lead straight punch to the head.
- The boxer describes how to perform the skill correctly.
- The boxer compares defensive techniques and the timing of when to use each.

Psychomotor LOs included:

- The boxer performs the skill correctly from stability in place while facing their teammate.
- In response to a coach signal, the boxer performs the skill correctly when moving and facing his teammate.
- In a conditional bout with his teammate for two minutes, the boxer performs the skill correctly.

Effective LOs included:

- The boxer cooperates with his teammate during the performance.
- The boxer shows courage during the bout.
- The boxer shows focus and emotional stability when performing their skills.

Select Strategies, Technology,

### 3. Select methods and media

**Fig 6:**
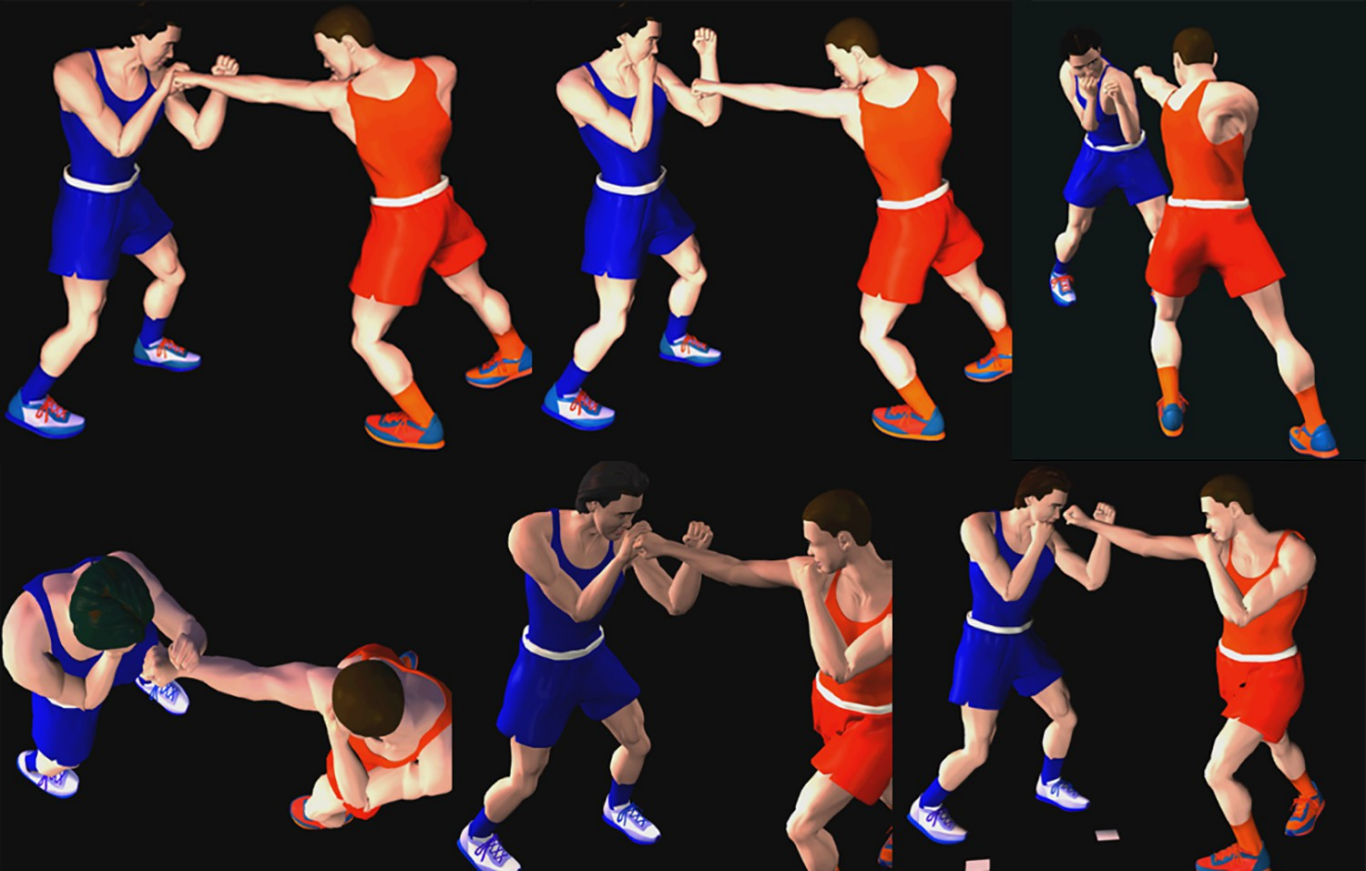
A collection of screenshots of defensive skills from 3D movies.

**Fig 7:**
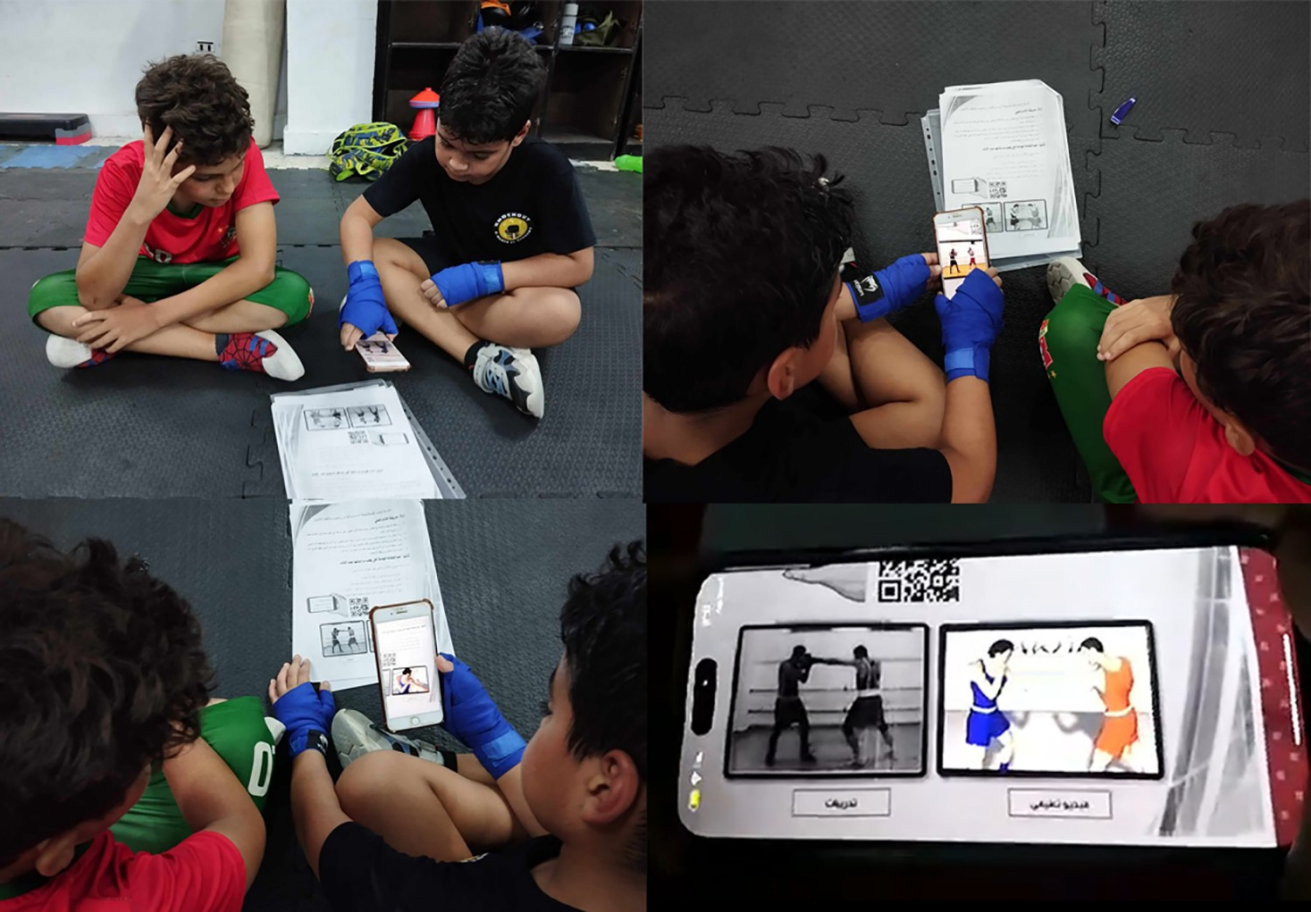
Boxers using AR technology as an example.

- *Media*: Rakha and Saleh [100] created 3D movies of fundamental defensive skills using Poser 7 software according to scientific standards. As an example, Fig 6 shows screenshots from these movies. A current study used these 3D movies to teach defensive skills through AR codes.
- *Design a printed guidebook for defensive boxing skills*: The defensive boxing skills under study are explained in a printed guidebook. Each skill is explained on one sheet of paper, followed by two images, one illustrating the skill and one illustrating exercises. Through AR codes, these images will be linked to 3D videos and appear to the learner as AR on the printed sheet.
- *Design AR-CODES*: For the creation of AR-Codes, a site called https://www.vidinoti.com/ was used, which offers five AR content free per account or through paid services. The Vidinoti services let users superimpose digital multimedia on top of real-world environments, and they access it through their smartphones or smart glasses. This allows for the creation of immersive experiences and interactive spaces. The platform https://armanager.vidinoti.com/ng2/home allows users to upload new content, and the Image AR tool was used to activate the marker-based AR system in this study, which links an image to a 3D movie. After selecting the image, the 3D movie is selected and placed on it. AR image is played through the following steps:
  - The V-Player app should be installed on the smartphone.
  - Access the V-director account page and scan the QR-code by using V-Player app to link the application to the digital content.
  - The V-Player app displays a message that content is available. When the boxer points the smartphone camera at the illustrated image in the guidebook, a 3D educational movie plays on the sheet as AR.
- *Methods*: A flipped classroom strategy was used to deliver educational program content via AR codes outside the club. Players can access 3D movies at anytime and anywhere by using a boxing defensive skills printed guidebook that includes AR codes. Once this has been completed, there is face-to-face interaction with the coach to implement applied learning activities during the formal session. Fig 7 shows a sample of Boxers using AR technology.

### 4. Utilize media and technology

There are two types of educational activities, one performed by the coach and one by the player:

- *Coach’s activities*: The coach explains the objectives of an educational module, delivers the guidebook to the players, determines a schedule for learning defensive skills, and motivates boxers to interact with AR technology before coming to the club. As part of club training sessions, boxers use AR-codes to show a 3D movie of the basic defensive skills that will be practiced, then the coach prepares players with warm-up and physical preparation exercises. In the next step, both boxers work together both offensively and defensively, taking into account how their roles change, while the coach guides them towards their goals.
- *The boxers’ activities*: Reading the guidebook and interacting with AR codes with a smartphone. In club training sessions, performs tasks under coach guidance, collaborates with his teammate and switches roles between offensive and defensive roles.

### 5. Require learner participation

The educational program is scheduled for five weeks, with three educational modules per week. The duration of the module is 90 minutes, and it is divided into the following sections:

**Table:** 

(a) *Experimental group*: Using the guidebook and interacting with the AR codes. *Control group*: Coach’s verbal illustration and modelling of the skill.	10 min
(b) General warm up	5 min
(c) Specific Physical Fitness	10 min
(d) Learning and Practice defensive skills	45 min
(e) Evaluation	10 min
(f) End	10 min

### 6. Evaluate and revise

The proposed program that uses AR codes was evaluated using a checklist for arbitrators to evaluate boxing defensive skills. Participants were also asked about their attitudes toward the program and their satisfaction with AR codes.

### The pre-measurements process

Pre-measurements were taken at Port Fouad Sports Club between 10-11/07/2023, with the assistance of four boxing coaches in order to confirm homogeneity and equivalence between the groups in terms of age, height, weight, general IQ, special physical abilities, and skill level in stance, footwork, and four straight punches. The homogeneity of the pre-measurements for each group is displayed in Table 2. Additionally, Table (3) displays equivalence for those variables between the two groups.

**Table 2:** Control and experimental groups’ descriptive statistics (*n*_1_ = *n*_2_ = 30).

Variables	Groups	*M*	*SD*	*Skew*	*SE*	*Kolmogorov-Smirnov*		
						*D*	*df*	*P*
Height (cm)	Experimental	151.90	4.99	.61	.43	.148	30	.09
	Control	149.63	5.71	.33	.43	.141	30	.13
Weight (kg)	Experimental	52.7	11.81	.41	.43	.118	30	.20
	Control	55.17	12.31	.19	.43	.096	30	.20
Age (y)	Experimental	12.61	.41	.36	.43	.126	30	.20
	Control	12.42	.38	.77	.43	.150	30	.08
IQ (Score)	Experimental	35.6	2.09	.43	.43	.152	30	.07
	Control	36.9	3.29	-.61	.43	.149	30	.09
Reaction Time (The ruler drop test) (cm).	Experimental	10.8	1.70	.07	.43	.148	30	.09
	Control	11.03	2.23	.39	.43	.145	30	.11
Kinetic velocity (Punch a heavy sandbag straight left and right for 10 seconds) (The maximum number of punches)	Experimental	30.87	3.59	.25	.43	.154	30	.06
	Control	29.17	3.89	.3	.43	.118	30	.20
Right straight punch power (Distance-throwing a 2kg medicine ball)(m)	Experimental	4.59	.46	-.05	.43	.113	30	.20
	Control	4.35	.57	-.58	.43	.106	30	.20
Left straight punch power (Distance-throwing a 2kg medicine ball)(m)	Experimental	4.02	.47	.33	.43	.119	30	.20
	Control	3.97	.58	.09	.43	.123	30	.20
Agility test (Quadruple jump in 10 sec)	Experimental	5	1.46	.07	.43	.153	30	.07
	Control	4.96	1.45	-.01	.43	.146	30	.10
Endurance in performance (Two-minute shadow boxing) (number of punches).	Experimental	129.5	4.56	-.06	.43	.108	30	.20
	Control	128.3	5.72	-.36	.43	.150	30	.08
Endurance of the muscles (Sit up from lying down) (The maximum number possible).	Experimental	32	2.86	-.20	.43	.136	30	.16
	Control	33.03	3.09	-.33	.43	.138	30	.15
Endurance of the muscles (Push-Ups) (The maximum number possible)	Experimental	13.30	3.65	1.12	.43	.139	30	.14
	Control	13.76	3.36	1.23	.43	.153	30	.07
Cardiopulmonary Endurance (Running 1500 m) (Time).	Experimental	6.5	.87	.34	.43	.135	30	.17
	Control	6.41	.73	.49	.43	.149	30	.09
Boxing Stance (Arbitrators’ mean scores)	Experimental	2.17	.51	.00	.43	.139	30	.15
	Control	2.11	.53	.24	.43	.150	30	.08
Footwork (Arbitrators’ mean scores)	Experimental	2.17	.51	.00	.43	.139	30	.15
	Control	1.91	.57	.14	.43	.132	30	.19
The mean of four Straight punches (Arbitrators’ mean scores)	Experimental	8.44	.45	-.11	.43	.111	30	.20
	Control	8.34	.49	-.09	.43	.072	30	.20

**Table 3:** Comparison of control and experimental samples using T-tests for the pre-measurements (*n*_1_ = *n*_2_ = 30).

Variables	Levene’s Test for Equality of Variances		t-test for Equality of Means				
	*F*	*p*.	*t*	*df*	*p*	Mean Difference	Std. Error Difference
Height (cm)	0.06	0.81	1.64	58	0.11	2.27	1.39
Weight (kg)	0.01	0.93	-0.79	58	0.43	-2.47	3.11
Age (y)	0.19	0.66	1.91	58	0.06	0.19	0.10
IQ (Score)	3.51	0.07	-1.82	58	0.07	-1.30	0.71
Reaction Time (The ruler drop test) (cm).	2.37	0.13	-0.46	58	0.65	-0.23	0.51
Kinetic velocity (Punch a heavy sandbag straight left and right for 10 seconds) (The maximum number of punches)	0.06	0.82	1.76	58	0.08	1.70	0.97
Right straight punch power (Distance-throwing a 2kg medicine ball)(m)	0.54	0.47	1.79	58	0.08	0.24	0.13
Left straight punch power (Distance-throwing a 2kg medicine ball)(m)	1.70	0.20	0.39	58	0.70	0.05	0.14
Agility test (Quadruple jump in 10 sec)	0.01	0.91	0.09	58	0.93	0.03	0.38
Endurance in performance (Two-minute shadow boxing) (number of punches).	1.75	0.19	0.90	58	0.37	1.20	1.34
Endurance of the muscles (Sit up from lying down) (The maximum number possible).	0.55	0.46	-1.34	58	0.18	-1.03	0.77
Endurance of the muscles (Push-Ups) (The maximum number possible)	0.22	0.64	-0.52	58	0.61	-0.47	0.91
Cardiopulmonary Endurance (Running 1500 m) (Time).	1.42	0.24	0.47	58	0.64	0.10	0.21
Boxing Stance (Arbitrators’ mean scores)	0.00	0.96	0.42	58	0.68	0.06	0.13
Footwork (Arbitrators’ mean scores)	0.08	0.77	1.83	58	0.07	0.26	0.14
The mean of four Straight punches (Arbitrators’ mean scores)	0.28	0.60	0.84	58	0.40	0.10	0.12

According to Table 2, the control group’s skewness ranged from -0.61 to 0.77 (*SE* = 0.43). The skewness values for the experimental group ranged from -0.20 to 0.61 (*SE* = 0.43). Because the two groups’ skewness values are less than the absolute value (1.96*.43 = .84), the normal distribution is verified [117,118]. Additionally, the Kolmogorov-Smirnov test was used for checking the normality of the pre-measurements in both the experimental and control groups. Test results varied between *D* (30) = .07, *P* = .20 and *D* (20) = .15, *P* = .06, exceeding the significance level (.05). Therefore, the null hypothesis was accepted, which means that the results were normally distributed in both groups. An independent samples t-test can be used to confirm the equivalence of the two groups [118].

The results of Levene’s test in Table 3 showed *F*-values ranging from (0) to (3.51), and *P*-values ranging from (.07) to (.96), which exceeded the significance level (.05), and hence the regular (*t*) and (*df*) values were used [119]. For all variables, the *t*-test values ranged from -1.82 to 1.91, and the *P*-values were greater than .05. It was accepted that the two groups are statistically equivalent in the variables of the pre-measurement, confirming that the null hypothesis is true [118].

### The basic experiment process

The experimental and control groups were coached three times per week by a boxing coach from the Port Fouad Sports Club in Port Said, Egypt, during the period between July 15 and August 18, 2023. Through a flipped classroom strategy, experimental group participants learned defensive skills under study through the proposed program with AR codes. Alternatively, the control group learned through command style, where the coach explained the skill, performed the model, and instructed the boxers to repeat it. Coach has ten years’ boxing experience and is certified by the Egyptian Boxing Federation. Moreover, he competed internationally as a boxer previously. The author conveyed the intended educational programme and timetable to him. As well as explaining how the same procedures may be applied to both groups. The author to ensure a satisfactory learning environment and to keep track of the progress of the basic experiment conducted a regular visit to the coach.

### The post-measurements process

On August 18, 2023, post-measurements were performed immediately after the basic experiment was completed. Three arbitrators applied the skill performance evaluation checklists [110] on the control and experimental groups and with care the same conditions of procedures, observation and application time.

### Statistical analysis

IBM SPSS Statistics for Windows (2017; version 25; IBM Corp, Armonk, NY, USA) was used for the following statistical analysis: Mean (*M*), Standard Deviation (*SD*), Skewness coefficient, Levene’s Test (*F*), Kolmogorov-Smirnov test (*D*), and Independent samples *t*-test.

## Results

### Research Question. What are the effects of AR-Codes on boxers’ performance of some fundamental defensive techniques?

#### First sub question: Is there a difference in post-measurement of certain fundamental defensive techniques between experimental and control groups

*H*_*a*_: There are statistically significant differences between the control and experimental groups in the post-measurements of some fundamental boxing defensive techniques in favour of the experimental group.

In Table 4, the experimental group’s Kolmogorov-Smirnov test for fundamental defensive skills ranged from *D* (30) = .14, *p* = 0.16 to *D* (30) = .16, *p* = 0.05. In the control group, the values ranged from *D* (30) = 0.13, *p* = 0.17 to *D* (30) = .16, *p* = 0.5. The null hypothesis was accepted because P-values greater than 0.05 indicated that the experimental and control groups followed a normal distribution. As a result, an independent samples t-test was used to determine whether any statistically significant differences existed between the groups. In addition, Levine’s test showed *F* values ranging from .00 to 2.40, and p values greater than .05, indicating that variances were not significantly different. As a result, the normal t value and degrees of freedom were used.

**Table 4:** Comparison of control and experimental groups using a t-test in the post-measurements (*n*_1_ = *n*_2_ = 30).

No	Defensive skills against straight punches under study	Groups	*M*	*SD*	Kolmogorov-Smirnov			Levene’s Test for Equality of Variances		Independent samples t-test		
					*D*	*df*	*p*	*F*	*P*	*t*	*df*	*P*
	**Left straight punch to the head (Jab)**											
1	Block punch with right hand—Push punch to internal with right hand	Experimental	8.27	.12	.15	30	.10	.56	.46	2.48	58	.02*
		Control	7.85	.11	.14	30	.13					
2	Leaning trunk to backward—Leaning trunk to right	Experimental	8.73	.14	.14	30	.16	.08	.78	2.68	58	.01*
		Control	8.16	.15	.16	30	.05					
3	Footwork to backward—Footwork to right	Experimental	8.71	.14	.16	30	.06	.09	.77	2.57	58	.01*
		Control	8.17	.15	.15	30	.08					
	**Right straight punch to the head**											
4	Block punch with right hand—Push punch to internal with left hand	Experimental	8.12	.12	.16	30	.05	.78	.38	2.09	58	.04*
		Control	7.78	.11	.14	30	.13					
5	Leaning trunk to backward—Leaning trunk to left	Experimental	8.57	.16	.15	30	.07	.56	.46	2.29	58	.03*
		Control	8.07	.15	.13	30	.17					
6	Footwork to backward—Footwork to left	Experimental	8.70	.16	.15	30	.11	.57	.45	2.45	58	.02*
		Control	8.15	.15	.14	30	.13					
	**Left straight punch to the trunk**											
7	Block by right forearm	Experimental	7.92	.12	.15	30	.09	2.40	.13	3.65	58	.00*
		Control	7.16	.17	.14	30	.15					
8	Footwork to backward- Footwork to right	Experimental	8.67	.16	.14	30	.11	.04	.83	2.94	58	.00*
		Control	7.98	.17	.15	30	.09					
	**Right straight punch to the trunk**											
9	Block by left forearm	Experimental	7.94	.13	.15	30	.10	2.21	.14	3.50	58	.00*
		Control	7.18	.17	.14	30	.11					
10	Footwork to backward- Footwork to Left	Experimental	8.64	.15	.15	30	.08	.00	.96	3.13	58	.00*
		Control	7.94	.16	.14	30	.14					
Overall average scores for skills		Experimental	8.48	.14	.11	30	.20	2.51	.12	3.07	58	.00*
		Control	7.92	.12	.14	30	.15					

*Reject the null hypothesis.

The experimental group’s mean defensive skills scores ranged between (*M* = 7.92, *SD* = 0.12) and (*M* = 8.73, *SD* = 0.14), which was greater than the control group’s mean defensive skills, which varied between (*M* = 7.16, *SD* = 0.17 and *M* = 8.17, *SD* = .15). The independent samples t-test indicated *t* (58) = 2.09 to 3.65 and *P*-values less than the significant level of .05. As a result, the null hypothesis was rejected, indicating that the experimental group outperformed the control group.

**Fig 8:**
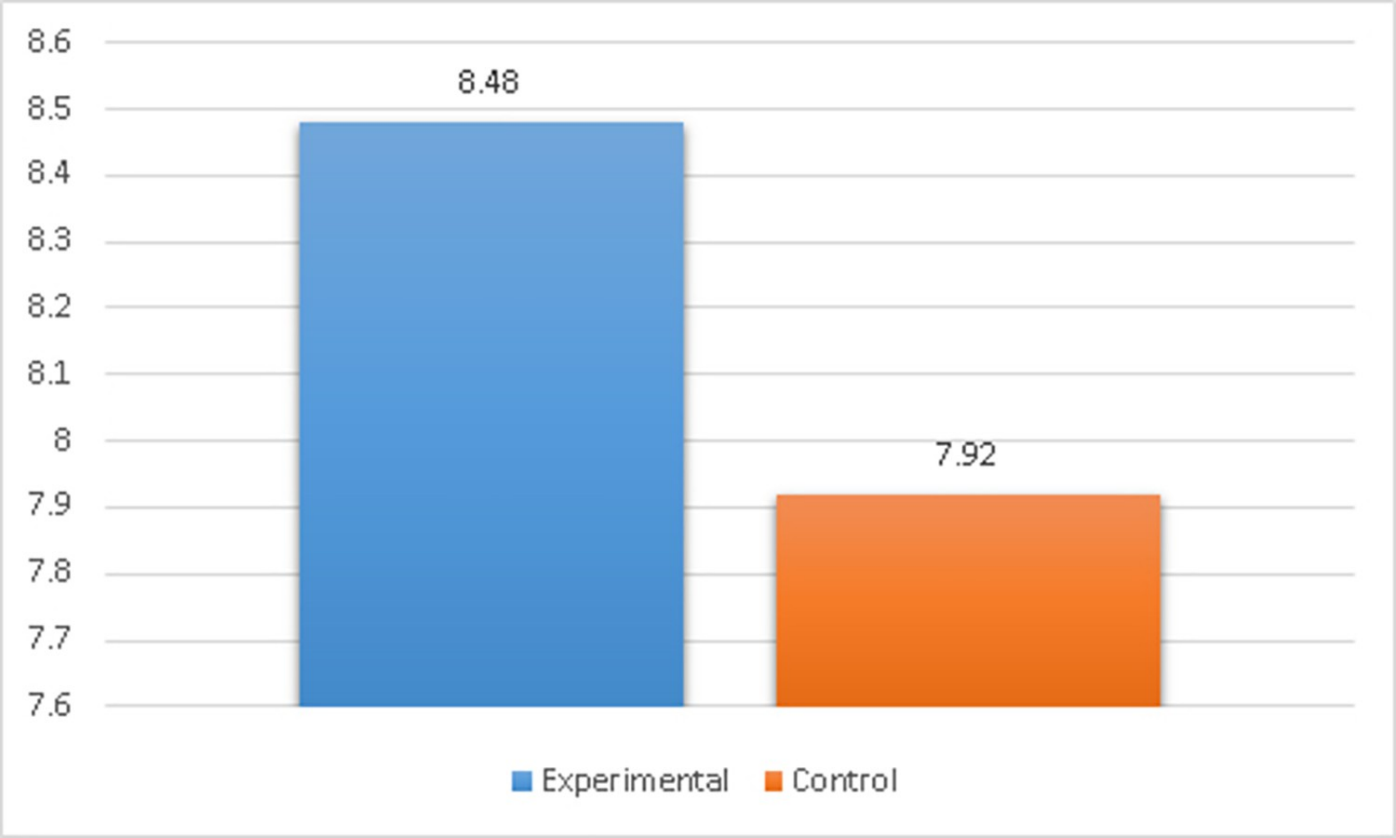
Comparison of defensive skill scores between experimental and control groups. The experimental group outperformed the control group in post-measurements of fundamental boxing defensive techniques.

Based on Table 4 and Fig 8, the overall skill performance score for the experimental group (*M* = 8.48, *SD* = .14) was higher than that of the control group (*M* = 7.92, *SD* = .12). There was statistically significant difference between the two groups, *t* (58) = 3.07, *p* ≤.01. Therefore, this null hypothesis was rejected. As a result, the experimental group outperformed the control group in the post-measurements of overall average scores for skills.

#### Second sub question: Is there a difference in post-measurement of certain fundamental defensive techniques between experimental and control groups

A higher overall skill performance score occurred in the experimental group (*M* = 8.48, *SD* = .14) than in the control group (M = 7.92, SD = .12). The effect size of the differences in the overall skill performance scores between the two groups at the post-test was (*η*^2^ = 0.14), which indicates a large effect size [105].

## Discussion

An educational programme based on AR-Code technology was created for instructing boxers some fundamental defensive skills against straight punches in a way that captured their attention, created tension, and demonstrated how it affected their performance.

Based on the findings of the first sub question, the experimental group performed superior to the control group in terms of the boxing fundamental defensive skills under study. This is consistent with several previous studies Baabdullah et al. [45]; dos Anjos et al. [56]; Khan et al. [55]; Papanastasiou et al. [49]; and Tiede et al. [50] which indicated that AR technology enhanced learners’ cognitive, skilful and emotional results. Thus, the proposed program, which incorporated AR technology into the educational program via the flipped classroom strategy, proved highly effective with the experimental group. With a marker-based AR system, the printed scientific materials in the boxer guidebook for defensive skills were enriched with 3D educational materials, improving knowledge and understanding of the sequence of motor performance of defensive skills, as well as enhancing motivation to learn. Moreover, the AR technology enabled the coach to engage the boxers and facilitate boxer-centered learning activities, promote self-learning, and enable live simulations and visualizations. This is consistent with the results of previous studies Chen and Liu [60]; and Quqandi et al. [61], which concluded that AR technology promotes student-centered learning and encourages self-learning by allowing students to access and interact with AR educational resources at the appropriate time and place for them.

Based on the findings of the second sub question, the proposed program incorporated with AR technology that used with the experimental group has a very large effect size. From this point of view, the ASSURE ID model enhanced the effectiveness of the proposed educational program. It improved the integration of AR technology into the program, based on boxers’ characteristics, as well as the selection of teaching and evaluation strategies that were appropriate for achieving the desired outcomes. The studies by Baran [92]; Kristianti et al. [94]; and Rim and Choi [93] indicated that teachers whose classrooms are designed according to the ASSURE model are more effective at integrating technology into the curriculum, and students’ critical thinking skills are significantly improved as a result of the ASSURE model, which improves the effect size of integrating technology.

With the flipped classroom strategy, boxers are able to learn on their own schedule and based on their own experiences with AR technology outside of training sessions. This increases the effectiveness of the contact hours in the club, as the boxer holds previous experience on the skill that will be learned during the club training session. Studies by Aburezeq [83], Fathi and Rahimi [87], Joseph et al. [86], Låg and Sæle [79], Sailsman [81], Webb and Doman [84], and Zheng et al. [85] have shown that flipped classrooms promote active learning during class contact hours, while encouraging independent learning outside the classroom. Moreover, students were able to learn material according to their own pace, schedule, and path through online learning platforms before attending face-to-face classes.

As part of mobile learning, QR-Codes give boxers the ability to link printed scientific materials in the Boxer’s Guidebook to AR digital educational resources via a smartphone connected to the Internet, making it easy to access digital educational resources and to display them according to the educational process. This is in line with the findings of studies by Chung et al. [69], Palazón and Giráldez [68], and Traser et al. [67], which indicated that QR codes could bridge the gap between printed and multimedia educational resources in education, thereby improving learning outcomes, learning effectiveness, and learners’ attitudes toward learning.

## Limitations

An important limitation of the present study is that AR-CODES requires a smartphone to connect to the Internet in order to access the interactive boxer’s guide with augmented reality technology, regardless of whether one is inside or outside the club. Three boxers without smartphones were verified and loaned tablets with data SIM cards so they could connect to the Internet and interact with AR-CODES. Another limitation of the study is that it consisted exclusively of male boxers, since there were only six female boxers, who were not beginners.

## Conclusions

The purpose of this study was to investigate the effectiveness of AR Codes technology on learning some basic boxing defensive skills. The study utilized a quasi-experimental method in which experimental and control groups were created. Experimental participants learned fundamental boxing defensive techniques using AR codes. On the other hand, the control group is taught a program based on the coach’s command style. A pre-post-test design was used for both groups. In post-measurement, the experimental group performed better than the control group. AR Codes technology had a significant impact on the learning of the boxing defensive skills under study. Consequently, it is necessary to use AR Codes technology as an educational resource to enhance the learning process, integrate it with active learning strategies, and use it to teach defensive boxing skills and apply them to offensive and counterattack skills, thereby improving the learning process. Further studies are needed to determine how to integrate AR Codes technology into education in conjunction with active learning strategies to teach different boxing skills and spread them to other sports and games. In addition, other types of AR technology can be used for education, such as position markers for teaching boxers how to use boxing tools and devices, or Boxing Circuit training for explaining each station.

## Supporting information

S1 Appendix An example of an educational module used by the experimental group. (PDF)

S2 Appendix An example of a defensive skill in the boxer’s guidebook that contains AR-Codes. (PDF)

S3 Appendix The checklist for arbitrators to evaluate the fundamental skills in boxing. (PDF)
