# Supplementary material for: Reflections on augmented reality codes for teaching fundamental defensive techniques to boxing beginners
Source: PLoS One. 2024 Apr 11;19(4):e0301728. doi: 10.1371/journal.pone.0301728 (PMC11008871; doi:10.1371/journal.pone.0301728)
Supplement: S1 Appendix — (PDF) [file pone.0301728.s001.pdf]

Appendix (1): A Sample of the Worksheet used in the First Lecture for the Experimental Group

The defensive skills by using arms against a straight left punch to the head (the Jap)

Lecture: One	Week: One	Date: / /	Subject: Technical:<br>Block with right hand (25 min)<br>Push to internal with right hand (20 min)

First: The introductory part of the training module

Subject	Description	Work amount				Notes
		counts	Performance time	Rest time	Total time (min)	
Theoretical lecture and Interaction with AR-Codes (10 min)	1- Block with right hand (5 min)<br>2- Push to internal with right hand (5 min)				10	
General warm up (5 min)	Running around the ring and performing general fitness drills to prepare the body				5	
Specific warm up (10 min)	(Standing. Hand on head side) Using your hand, alternately push the head to the side with resistance.				1	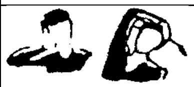
	(Lie down with your hands below your shoulders) Extend your arms while raising your torso.	3	30 sec	30 sec	3	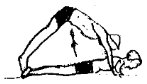
	(Sitting on all fours) jumping up with arms raised high.	3	30 sec	30 sec	3	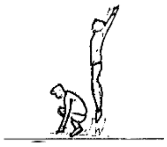
	(Standing, holding a 5 kg medicine ball) Throw the ball up with one hand.	3	30 sec	30 sec	3	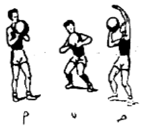

Second: The practice and exercises of defensive skills

Subject	Description	Work amount				Notes
		counts	Performance time	Rest time	Total time (min)	
Technique (45 min)	**<u>A) Block with right hand (25 min):</u>**<br>1- The boxer perform the defense skill alone while imagining the opponent punching him.	10			5	
	2- With coach's signal, the defending boxer confronts the colleague, the colleague executes the punch in two counts (1) for half the distance, (2) for the complete distance, and the defender executes the defensive skill, and then returns to the stand-by position.	10			5	
	3- Perform the same exercise as before in one count after the coach's signal	10			5	
	4- Without the coach's signal, perform the same exercise from stance in one count.	10			5	
	5- Freely perform the defense skill with an offensive colleague when he move	10			5	

	**<u>B) Push to internal with right hand (20 min):</u>**<br>1- With coach's signal, the defending boxer confronts the colleague, the colleague executes the punch in two counts (1) for half the distance, (2) for the complete distance, and the defender executes the defensive skill, and then returns to the stand-by position.	10			5	
	2- Perform the same exercise as before in one count after the coach's signal<br>3- Without the coach's signal, perform the same exercise from stance in one count.<br>4- Freely perform the defense skill with an offensive colleague when he move	10<br>10<br>10			5<br>5<br>5	
Evaluation (10 min)	Assessing defensive skill and providing feedback to boxers using the checklist					
Cool down (10 min)	1. Shadowboxing for 2 min of low-intensity, very slow shadowboxing in front of a mirror.<br>2. Static Stretching (Shoulder Pull, Triceps Pull, Chest Pull, Standing Quad Pull)				2<br>8	
