# Supplementary material for: Reflections on augmented reality codes for teaching fundamental defensive techniques to boxing beginners
Source: PLoS One. 2024 Apr 11;19(4):e0301728. doi: 10.1371/journal.pone.0301728 (PMC11008871; doi:10.1371/journal.pone.0301728)
Supplement: S2 Appendix — (PDF) [file pone.0301728.s002.pdf]

Dear Boxer:

Utilizing your smartphone, you can enhance your understanding of the scientific material printed in this guide by using AR-Code technology. To allow you to see the pictures printed in this guide moving and explaining how to perform the skill, as well as how to perform exercises to master it with a colleague**.**

Follow these steps to activate the AR-Code technology:

1- You can download the V-Player app by scanning the QR-code below:

2- Use the V-Player app to scan the QR-Code accompanying each image, and then point your camera at the image you want to view.

Note: All defensive techniques have been explained for right-handed boxers.

Block with right hand against straight left punch to the head

How to perform the skill?

1. A boxer's stance from a long punching distance in confronting their opponent. It refers to the distance you have between you and your opponent when you extend your arm.
2. An opponent performs the left straight punch to the head until the middle of the long punching distance. Your right hand fist begins to open.
3. The opponent extends his left arm in order to complete the punch. Extend your right fist and twist it outward in front of your chin no more than 3 cm until the opponent's fist is blocked.
4. You and your opponent should go back to the boxing stance.

There are some important rules that boxers must follow:

1. Make sure your left arm is maintain in a ready position
2. You should look at your opponent's body to anticipate what he will do next.
3. Right arm muscles contract in proportion to punch force
4. A right elbow is attached to the trunk in order to protect and cover it.

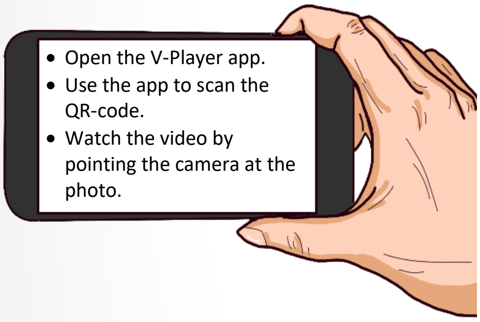

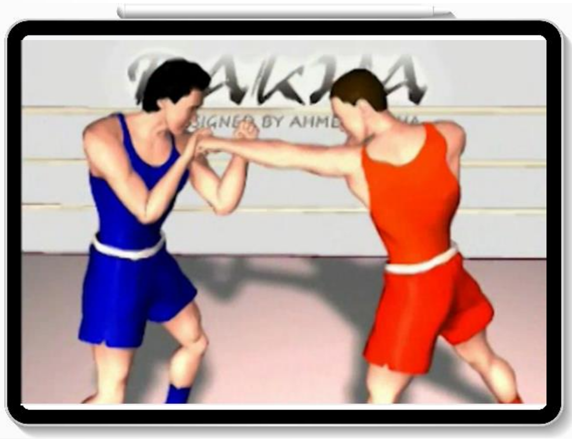

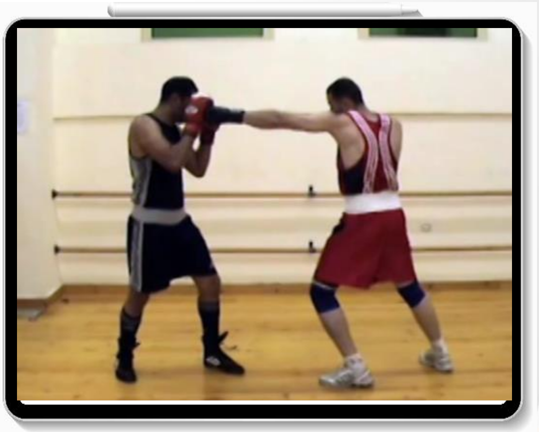

An educational 3D movie

Exercises
