# Supplementary material for: Reflections on augmented reality codes for teaching fundamental defensive techniques to boxing beginners
Source: PLoS One. 2024 Apr 11;19(4):e0301728. doi: 10.1371/journal.pone.0301728 (PMC11008871; doi:10.1371/journal.pone.0301728)
Supplement: S3 Appendix — (PDF) [file pone.0301728.s003.pdf]

Appendix 2. The Skill performance evaluation checklists by arbitrators

First: Technical criteria for evaluating skills

	Technical criteria	Ideal degree of performance
1	Stance	
	- Maintaining the correct head position.	1
	- Maintaining the correct position of arms and fists.	1
	- Rotation of the trunk with a slight forward tilt.	1
	- Bending knees and distributing body weight on feet	1
	- Standing on back foot instep.	1
	Total degree for stance	5
2	Foot work	
	- Keeping forward and backward distances as possible.	1
	- Keeping side distance as possible.	1
	- Moving on insteps.	1
	- Crawling correctly with the foot	1
	- Ideal speed for performance.	1
	- total degree for foot work	5
3	Straight left to head	
	- Extending left arm for the required distance.	2
	- Continuing performance by moving body weight.	1
	- Pelvis and shoulder rotation to the right	1
	- Return of left arm at the same kinetic path for best performance	2
	- Right arm and fist in defense position during performance	1
	- Ideal speed for performance.	2
	- Total shape of performance	1
	- Total degree for straight left to head.	10
4	Straight left to Trunk	
	- Extending left arm for the required distance.	2
	- Bending knees and leaning with trunk to the right	1
	- Pelvis and left shoulder rotation to the right	1
	- Return of left arm at the same kinetic path for best performance	2
	- Right arm and fist in defense position during performance	1
	- Ideal speed for performance.	2
	- Total shape of performance	1
	- Total degree for straight left to Trunk.	10
5	Straight right to head	
	- Extending right arm for the required distance.	2
	- Continuing performance by moving body weight.	1
	- Pelvis and shoulder rotation to the right	1
	- Return of right arm at the same kinetic path for best performance	2
	- Left arm and fist in defense position during performance	1
	- Ideal speed for performance.	2
	- Total shape of performance	1
	- Total degree for straight right to head.	10
6	Straight right to trunk	
	- Extending left arm for the required distance.	2

	- Bending knees and leaning with trunk to the left	1
	- Pelvis and right shoulder rotation to the left	1
	- Return of right arm at the same kinetic path for best performance	2
	- Right arm and fist in defense position during performance	1
	- Ideal speed for performance.	2
	- Total shape of performance	1
	- Total degree for straight right to trunk.	10
	Defensive skill	
	Correctly move the part to be defended against the punch (arms, trunk, legs)	2
	Performing the defense while maintaining the form of a ready stance	2
7	In response to the punch, the defensive skill is executed at the required speed	2
	To counterattack, the boxer returns to a ready stance and maintains the appropriate punching distance	2
	Looking at the opponent while defending with the head up	2
	- Total degree for the defensive skill	10

Second: phases of evaluating skills

Basic skills under investigation.		Phases of evaluating performance.
1	Stance.	Performing skill from still in slow counts with stability in performance
2	Foot work.	Performing skill in slow counts and return to stance.
3	Attack skills.	Performing skill from movement (Shadow boxing).

Third: Evaluation checklists for performing the skills under investigation

Skills		Ideal degree	Student's degree
**Basic skills**	Stance.	5	
	Foot work.	5	
**Attack skills**	Straight left to head	10	
	Straight left to torso	10	
	Straight right to head	10	
	Straight right to torso	10	
**Defensive Skills**			
Straight left to head	Block with right hand	10	
	Push to internal with right hand	10	
	Leaning trunk to backward	10	
	Leaning trunk to right	10	
	Footwork to backward	10	
	Footwork to right	10	
Straight left to trunk	Block by right forearm	10	
	Footwork to backward	10	
	Footwork to right	10	
Straight right to head	Block with right hand	10	
	Push to internal with left hand	10	
	Leaning trunk to backward	10	
	Leaning trunk to left	10	

	Footwork to backward	10
	Footwork to left	10
Straight right to trunk	Block by left forearm	10
	Footwork to backward	10
	Footwork to right	10
Total for performance level		230
